# NURSING HOMES’ FIVE-STAR QUALITY RATING SYSTEM: FORECASTING THE FUTURE STABILITY AND VOLATILITY

**DOI:** 10.1093/geroni/igad104.2335

**Published:** 2023-12-21

**Authors:** Shivani Gupta, Ashay Kadam, Neeraj Bhandari

**Affiliations:** University of Houston - Clear Lake, Houston, Texas, United States; Goa Institute of Management, Sattari, Goa, India; University of Nevada, Las Vegas, Las Vegas, Nevada, United States

## Abstract

The utilization of publicly reported quality measures such as nursing homes’ five-star quality rating system to hold providers accountable for their quality of care has increased over the years. Therefore, nursing homes have invested significant resources into maintaining or improving the ratings awarded to them by CMS. This study utilizes statistical models to analyze the past rating histories to predict future evolution of ratings of nursing homes. Data on all U.S. nursing homes for the years 2017 - 2021 was derived from CMS Nursing Home Compare. Primary variable represents the overall five-star quality rating of nursing homes. A Markov chain model and a Mover Stayer model are used to predict changes in ratings of nursing homes. The one-year rating transition probabilities are estimated using maximum likelihood estimation. The key findings indicate the likelihoods of future upgrades and downgrades in nursing homes’ ratings. Nursing home administrators are under constant pressure to strategically allocate resources for maintaining or improving the five-star ratings awarded to them by CMS. The findings of this study could help nursing home administrators in forecasting future change in their rating and making better resource allocation decisions such as those related to preventing a rating downgrade. The study findings could also be used by policymakers to focus on nursing homes facing downgrades and assisting them in improving their quality of service based on the potential change in their rating. The same results could also be utilized to assess the stability of ratings given to nursing homes.

